# Genetic and Epidemiological Evidence of Avian Influenza A(H9N2) Detection Among Poultry in Ghana, 2022

**DOI:** 10.3390/v18070725

**Published:** 2026-06-30

**Authors:** Stephen Ofori Nyarko, Lorreta Kwasah, Linda Boatemaa, Nana Afia Asante Ntim, Mildred Adusei-Poku, Gifty Mawuli Sarpong, Vanessa Magnusen, Jennifer Wutsika, Samuel Ago, Esinam Aku Apefa Amenuvor, Juliet Wordui, Ama Nyansema Sekyi-Yorke, Cecilia Takyi, Roberta Tackie, Innocent Kwao Doku, Joseph Asuam Nyarko, Joseph Ahia Quarcoo, Grace Arezie Kyiire, Theophilus Odoom, Fenteng Danso, William Asiedu, Daniel Lartei Mingle, Naiki Attram, Shirley Cameron Nimo-Paintsil, Sanders Terrel, Hugo Miranda Quijada, William Kwabena Ampofo, Ivy Asantewaa Asante

**Affiliations:** 1 Noguchi Memorial Institute for Medical Research (NMIMR), University of Ghana, Legon, Accra P. O. Box LG 581, Ghana; snyarko@noguchi.ug.edu.gh (S.O.N.); lkwasah@noguchi.ug.edu.gh (L.K.); lboatemaa@noguchi.ug.edu.gh (L.B.); nntim@noguchi.ug.edu.gh (N.A.A.N.); vmagnusen@noguchi.ug.edu.gh (V.M.); jwutsika@noguchi.ug.edu.gh (J.W.); sago@noguchi.ug.edu.gh (S.A.); jwordui@noguchi.ug.edu.gh (J.W.);; 2 West African Centre for Cell Biology of Infectious Pathogens (WACCBIP), University of Ghana, Legon, Accra P. O. Box LG 54, Ghana; 3 Department of Medical Microbiology, University of Ghana Medical School, Korle Bu, Accra P. O. Box LG 25, Ghana; 4 Department of Medical Biochemistry, University of Ghana Medical School, Korle Bu, Accra P. O. Box 4236, Ghana; 5 Veterinary Services Directorate, Accra P. O. Box M 161, Ghana; theodoom@yahoo.com (T.O.);; 6 Public Health Division, 37 Military Hospital, Accra P. O. Box BC 254, Ghana; 7 United States Naval Medical Research Unit-EURAFCENT, Ghana Detachment, 24 Fourth Circular Road, Cantonment, Accra P. O. Box GP 2288, Ghana

**Keywords:** AH9, prevalence, avian influenza, Ghana, poultry

## Abstract

Avian influenza viruses continue to pose significant zoonotic and pandemic threat globally, with low-pathogenic avian influenza A(H9N2) being of particular concern due to sustained circulation in poultry, adaptability, and repeated human spillover. This study investigated the detection and genetic characterization of influenza viruses at the animal–human interface in Ghana in 2022, using a nationwide cross-sectional One Health approach. Samples were collected from poultry, pigs, the environment, and animal handlers across backyard farms, commercial farms, and live bird markets. Laboratory testing was conducted using real-time RT-PCR, while statistical associations were assessed using chi-square and logistic regression. Whole-genome sequencing and phylogenetic analysis were performed on selected positive samples. Out of 4056 samples, 1516 were poultry samples. A(H9N2) was detected exclusively in poultry, with a prevalence of 5.67%. The Northern belt recorded the highest prevalence. Live bird markets had significantly higher odds of A(H9N2) detection compared with commercial farms (odds ratio: 15.37, *p* < 0.0001), while backyard farms had lower odds. Environmental samples were negative. Among animal handlers, one case each of A(H3N2) and SARS-CoV-2 was identified. Phylogenetic analysis demonstrated that Ghanaian strains belonged to clade G1 and possessed mammalian-adaptive markers. These findings highlight ongoing circulation in poultry and the need for sustained One Health surveillance.

## 1. Introduction

Avian influenza viruses (AIVs) have, in recent times, gained attention due to their sustained spread among wild birds [1]. Although the viruses are of avian origin, they are not bound by host incompatibility and geographic restrictions [2,3,4]. In birds, AIVs, in addition to causing respiratory distress, also cause systemic diseases that increase the risk of transmission to scavengers (such as foxes) and humans, particularly during meat preparation or farming [5,6,7]. The wide host range compatibility is primarily due to virus adaptability resulting from genetic mutations and reassortments with other influenza A subtypes [1].

AIVs are classified as either low pathogenic (LPAIV) or highly pathogenic (HPAIV) based on associated mortality in susceptible hosts and specific genetic variations [8,9]. While HPAIVs have gained significant attention due to their devastating effects among domestic poultry, monitoring LPAIV, particularly A(H9N2) viruses, remains crucial. Since its emergence in Asia, LPAIV A(H9N2) has spread worldwide, evolving into several distinct clades that sometimes overlap in specific geographic locations. Studies have identified five distinct clades: American, Eurasian (Y439), G1-W, G1-E, and BJ94 [10,11]. The American clade is primarily confined to the Americas, whereas the Eurasian, G1-E, G1-W and BJ94 clades cover Europe and Asia, with the Eurasian clade also detected in Australia [10,11]. In Africa, G1-W is the circulating clade in Western and Northern regions, while the Eurasian clade is predominant in Southern and Central Africa [11]. LPAIV A(H9N2) viruses have been isolated in multiple African countries, with Egypt serving as an endemic hotspot and frequent detection in poultry from Libya and Tunisia [12,13,14]. Furthermore, since 2016, the virus has been isolated in several North and West African countries, including Morocco, Burkina Faso, and Algeria, as well as in East Africa, notably in Uganda [11,15,16]. In Ghana, LPAIV A(H9N2) has been detected among poultry since 2017. These viruses were initially detected among bird populations kept on farms at military barracks. Although these viruses were LPAIV, genetic analyses showed mammalian adaptive motifs [17]. In 2019, the Veterinary Services Directorate in Ghana confirmed the presence of LPAIV A(H9N2) in poultry between November 2017 and February 2018, coinciding with farmers’ reports of a dramatic increase in mortality rates and a significant decline in egg production across several layer hen farms. Whole-genome sequencing confirmed these viruses were of the G1 lineage [18].

The sustained global threat of these viruses highlights the significance of continuous surveillance and research. Pandemic preparedness remains essential to mitigate the potential impact of future emerging infectious diseases. One key area of focus is the animal–human interface, where a One Health approach is utilized. This strategy, which recognizes the interconnectedness of human, animal, and environmental health, provides a strong foundation for pandemic preparedness efforts. During the COVID-19 pandemic in 2021, several regions in Ghana faced outbreaks of avian influenza among poultry. In response, surveillance activities incorporated a One Health approach and intensified nationwide avian influenza surveillance efforts. In 2022, similar outbreaks prompted the activation of the established One Health framework. The objective of these activities was to assess the regional distribution of avian influenza viruses, characterize them genetically, and investigate potential mammalian adaptations. This study presents the findings derived from the enhanced surveillance conducted using a One Health approach.

## 2. Materials and Methods

### 2.1. Study Design, Settings, and Population

**Figure 1:**
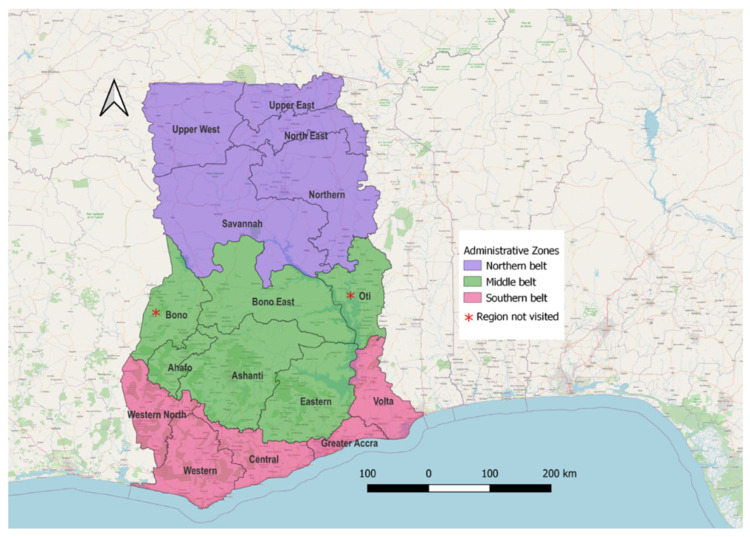
The 16 administrative zones in Ghana are geographically divided into: the Northern zone (purple), which comprises 5 regions (Upper East, Upper West, North East, Northern and Savannah), the Middle belt (green), which includes 6 regions (Ahafo, Ashanti, Bono, Bono East, Eastern and Oti) and the Southern belt (pink), comprising 5 regions (Central, Greater Accra, Volta, Western and Western North regions). Asterisks (*) represent regions where samples were not collected. Map of Ghana was obtained from OpenStreetMap (https://www.openstreetmap.org; 10 January 2023) and modified using Quantum Geographic Information System (QGIS) version 3.40.

This study was conducted using a cross-sectional design. From November to December 2022, coinciding with Ghana’s dry season, a nationwide sampling initiative was carried out in 14 of the 16 administrative regions of the country (Figure 1). The two exempted regions were Bono and Oti, because, before this study, the terrains had not been assessed, and there were no designated focal people identified to provide site-specific operational guidance. Due to the zoonotic potential of avian influenza viruses, this study utilized a One Health approach by sampling animals (birds and pigs), the environment (water samples from Ramsar wetland sites and estuaries) and humans (animal handlers on farms and live bird markets). We stratified the animal and farmers/live bird market workers/animal handlers sampling area into three sample groups: commercial farms, live bird markets (LBM), and backyard farms. A commercial farm was defined as a place whose primary focus was on the breeding, raising, and production of birds or pigs. An LBM was defined as a place (having ≥2 stalls) where live birds or pigs were brought for sale to consumers and businesses, while a backyard farm was defined as a person’s yard, where the breeding, raising, and production of birds or pigs were for family consumption. Specimens were collected as follows: tracheal and cloacal samples from birds; nasal and anal samples from pigs; water, feathers, and bird droppings from Ramsar sites as environmental specimens; and oropharyngeal and nasopharyngeal specimens from farmers/LBM workers/animal handlers. Out of Ghana’s six designated Ramsar sites, five were utilized as sampling stations.

### 2.2. Sampling Strategy, Sample Storage and Transportation

Animal sampling focused primarily on sick and dead animals, and all sick and dead birds were sampled. However, when present, healthy birds were sampled prior to sick birds. For healthy animals, a random sampling strategy was employed. For environmental sampling, all five selected sites were sampled. Samples were collected in 1 mL of in-house prepared viral transport medium (VTM), whereas environmental samples were collected into sterile 50 mL tubes. VTM was prepared as follows: In 1000 mL of sterile distilled water, 20 g veal infusion broth (Sigma-Aldrich, Darmstadt, Hesse Germany) and 4 g Bovine Albumin Fraction V (7.5%) (ThermoFisher Scientific, Waltham, MA, USA) were added. Subsequently, VTM was supplemented with 1.6 mL of gentamicin sulfate solution (100 mg/mL) (Life Technologies Limited, Waltham, MA, USA) and 6.4 mL of amphotericin B (250 µg/mL) (ThermoFisher Scientific, Waltham, MA, USA). This mixture was then filtered using a 0.22 µm pore-size (Sigma-Aldrich, Darmstadt, Hesse, Germany) membrane filter. All farmers/LBM workers/animal handlers who consented to take part in this study were sampled. To maintain sample integrity, collected samples were stored at ≤+4 °C using ice packs for short deployments and liquid nitrogen for long deployments. Samples were triple-packaged and cold-chain maintained during sample transportation to the Noguchi Memorial Institute for Medical Research (NMIMR), in the Greater Accra region, for analyses.

### 2.3. Virus Detection by Real-Time Reverse Transcription Polymerase Chain Reaction Assays (rRT-PCR)

The specimens were processed at the NMIMR in specialized Biosafety Level 3 (BSL-3) laboratories designated for animal work. For the animal swab, bird dropping, and feather samples, viral ribonucleic acid (RNA) was isolated using the QIAamp viral RNA mini kit (Qiagen, Hilden, North Rhine-Westphalia, Germany) following the manufacturer’s instructions. Before RNA isolation, the bird dropping samples were processed by homogenizing 1 g of sample in 5 mL of VTM, followed by centrifugation to remove particulate matter. About 2 cm from the calamus of the feather is used for the RNA isolation step, and these samples are processed individually. Feather samples were immersed in VTM for 48 h to facilitate the release of viral particles into the solution before RNA isolation. For the environmental water samples, 1.5 L of water was collected from designated Ramsar sites and processed individually using the two-phase separation method. For the two-phase separation method, samples were equilibrated at room temperature for 5 min to allow for sedimentation of the coarse material, after which 500 mL was centrifuged at 1500× *g* for 20 min at 4 °C, with the supernatant collected and the pellet stored at 4 °C. The supernatant was adjusted to pH 7.0–7.4 and supplemented with PEG6000 (Sigma-Aldrich, Darmstadt, Hesse, Germany), dextran (Sigma-Aldrich, Darmstadt, Hesse, Germany), and NaCl, followed by continuous stirring for 1 h. The mixture was transferred into sterile separation funnels and incubated overnight at 4 °C to allow for phase separation. The lower phase and interphase were collected, while the pellet was resuspended in concentrate, combined with ethanol-stabilized chloroform and sterile glass beads, and then shaken vigorously for 20 min. A final centrifugation at 1500 rpm for 20 min at 4 °C yielded the upper aqueous phase and interphase, which were collected into sterile tubes for downstream analysis, without antibiotic treatment [19]. Initial screening for influenza A viruses (IAVs) was done on both animal and environmental samples, with positive samples subsequently subtyped. These samples were analyzed using real-time reverse transcription polymerase chain reaction assays (rRT-PCR) with the AgPATH One-step RT-PCR kit (Thermofisher Scientific Inc., Waltham, MA, USA) and specific primers and probes, as described by the US Centers for Disease Control and Prevention (U. S. CDC) [20]. Samples collected from animal handlers were initially screened for both influenza and SARS-CoV-2 simultaneously and subtyped for influenza A (H3/H1/pd09) and influenza B lineage Victoria and Yamagata. Samples with cycle threshold (Ct) values < 40 were considered positive for the specified target, while samples with Ct values > 40 were considered negative for the specified target. For quality control and assurance purposes, nuclease-free water was included as a negative extraction control to monitor potential contamination during the RNA extraction process. Positive controls for the rRT-PCR assay consisted of U. S. CDC influenza A/H5, A/H7, and A/H9 control materials obtained from the International Reagent Resource, and a non-template control was used as the negative control [21]. These controls were included in each assay run to ensure accuracy and reliability of the rRT-PCR results.

### 2.4. Sequencing

A nanopore-based method was used to amplify and sequence the full genomes of LPAIV A(H9N2) viruses with Ct values below 30. The RNA isolation protocol of the QIAamp viral RNA mini kit (Qiagen, Hilden, North Rhine-Westphalia, Germany) was modified by using 280 μL of starting material to isolate 35 μL of RNA. All gene fragments of detected viruses were amplified using a combination of universal and gene-specific primers in PCR. The resulting PCR amplicons were confirmed on a 1% agarose gel and then purified with Agencourt AMPure XP beads (Beckman Coulter Inc., Brea, CA, USA). Full-genome sequences were generated using the Mk1B MinION platform with R9.4.1 flow cells, SQK-LSK109 Ligation Kit, (Oxford Nanopore Technologies, Oxfordshire, UK) and a native barcoding kit according to the manufacturer’s instructions [22].

### 2.5. Data Analysis

Data were cleaned by checking for errors, duplicates, and inconsistencies. Outcome variables were influenza virus subtypes or respiratory pathogens detected: influenza in animals and environmental samples, and influenza and SARS-CoV-2 in humans. Independent variables of interest included the type of farms, the type of animal, health status, and regional distribution. These data sets were analyzed into tables, expressed in frequencies and proportions. Chi-square test was used to measure association, and variables showing significant associations were included in the final model. Using logistic regression, univariate analysis was conducted, using a 95% confidence interval (CI) and an alpha level of 5% to estimate the odds ratio between the independent variables (study setting, bird type, and administrative zone) and influenza A(H9N2) as the outcome variable. To evaluate potential bias, a comparative analysis was performed to assess differences in characteristics between observations with and without missing values. In addition, a sensitivity analysis was conducted to examine the impact of missing data on this study’s results. Sequences were analyzed, and phylogenetic relationships were determined using Clustal Omega alignments and maximum likelihood (ML) methods in MEGA11 version 11.03.13. The robustness of the ML tree was assessed using 1000 bootstrap replicates. The evolutionary distances were calculated using the General Time Reversible method. Genetic analyses were done in Biological Sequence Alignment Editor (BioEdit) version 7.2.57 [23].

### 2.6. Ethics

This surveillance for avian influenza among domestic and wild bird populations in Ghana was conducted under emergency operations using a One Health approach. The US Naval Medical Research Command Institutional Review Board—Office of Research Administration (NAMRU3-PJT-21-01) determined that surveillance for respiratory pathogens falls under public health surveillance and, therefore, ethical review is not required. All procedures were performed according to Ghana’s guidelines and regulations. No administrative permissions were required to access data. Data were anonymized before use and only laboratory identities were used during analysis.

## 3. Results

Over a 2-month period, 14 out of 16 regions were visited, sampling 127 backyard farms, 60 commercial farms, and 74 LBMs. A total of 4056 samples were collected. Of these, 3032 were tracheal/cloacal specimens from 1516 birds, 222 were nasal/anal swabs from 111 pigs, 44 were environmental specimens (including bird droppings, water and feathers) from six Ramsar sites, and 758 were oro/nasopharyngeal specimens from 379 farmers/LBM workers/animal handlers. None of the animals sampled were vaccinated against any of the avian influenza subtypes.

### 3.1. Demographic Characteristics

Among the sampled animals, 67.48% (1098/1627) were fowl, 10.02% (163/1627) ducks, 8.23% (134/1627) guinea fowl, 4.24% (69/1627) other birds, 3.2% (52/1627) turkey, and 6.82% (111/1627) swine. The proportion of LPAIV A(H9N2) detection was highest in guinea fowl [11.98%, (16/134)], followed by fowls [5.83%, (64/1098)] and ducks [3.07%, (5/163)], while the lowest detection rate was observed in turkey [1.45%, (1/69)]. We sampled healthy [89.18%, (1451/1627)] and sick birds [10.69%, (174/1627)]. The proportion of LPAIV A(H9N2) detection among healthy and sick bird populations was 5.31% (77/1451) and 5.17% (9/174), respectively. Prevalence of LPAIV A(H9N2) was higher in LBMs [25%, (74/296)], followed by commercial farms [2.12%, (9/424)], and backyard farms [0.33%, (9/907)]. Additionally, 111 pigs were sampled, and none tested positive for influenza. The overall prevalence of LPAIV A(H9N2) detected in birds was 5.67% (86/1516). Chi-square and Fisher’s exact analysis revealed that the type of bird, farm settings, and administrative zones were associated with LPAIV A(H9N2) infection (*p* < 0.05). None of the environmental and farmers/LBM workers/animal handlers’ specimens tested positive for LPAIV A(H9N2) or any other AIVs. However, a positive case of influenza AH3 and SARS-CoV-2 each was detected among asymptomatic farmers/LBM workers/animal handlers (Table 1).

### 3.2. Logistic Regression Analyses

Following regression analysis of three variables (farm setting, bird type, and administrative zone) associated with LPAIV A(H9N2) infections, only one variable (farm setting) exhibited statistical significance. Using live commercial farms as a reference, it was observed that the likelihood of LPAIV A(H9N2) infection among poultry was 15.3704 for LBMs and 0.1530 for backyard farms (Table 2).

### 3.3. Geographical Distribution of LPAIV A(H9N2) Detected in Ghana, 2022

**Figure 2:**
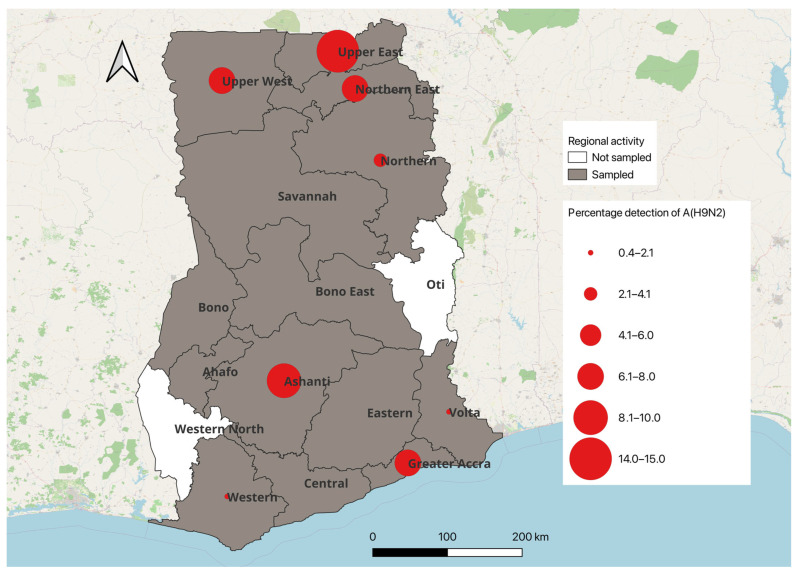
Visual representation of the areas covered during the sampling exercise, along with the proportion of specimens that tested positive for influenza A(H9N2). The light purple regions indicate areas where specimens were collected, while the white regions were not visited during the sampling activity. Map of Ghana was obtained from OpenStreetMap (https://www.openstreetmap.org; 10 January 2023) and modified using Quantum Geographic Information System (QGIS) version 3.40.

Of the five regions in the Northern belt, four reported LPAIV A(H9N2)-positive birds (Northern [4%, (6/151), North East [7%, (3/43)], Upper West [7.1%, (10/141)], and Upper East [14.4%, (21/146)]). In the Middle belt, five of six regions were included in the sampling activity; however, positive detections were observed only in Ashanti [9.7%, (18/185)]. In the Southern belt, three of the four sampled regions recorded LPAIV A(H9N2) cases, with the highest proportion in Greater Accra [6.6%, (24/365)], followed by the Western [2.0%, (3/149)] and Volta [0.4%, (1/231)] regions (Figure 2). Although the Southern belt accounted for the largest number of specimens collected, it recorded the lowest proportion of LPAIV A(H9N2) [3.61%, (28/775)] compared with the Northern belt [7.68%, (40/521)] and the Middle belt [5.44%, (18/331)].

### 3.4. Phylogenetic Analysis of Isolated H9N2 Sequences

**Figure 3:**
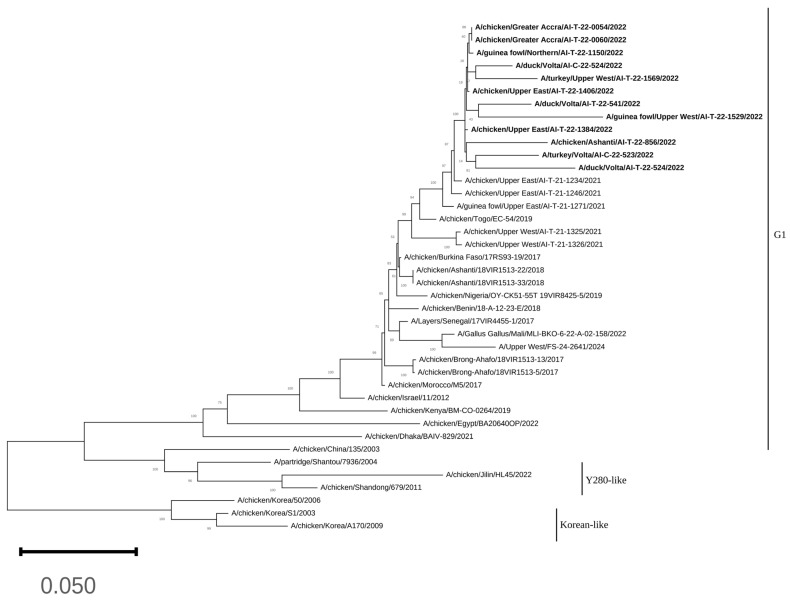
Phylogenetic analysis of the HA gene of the A(H9N2) avian influenza virus isolated from domestic poultry (chicken, turkey and guinea fowl) in Ghana. The viruses from this study are shown in bold. The posterior numerical and alphanumeric values indicate the clade designation of the HA gene.

Of the 86 LPAIV A(H9N2)-positive samples identified, 18 viruses were successfully sequenced, including one from Ashanti, two from Greater Accra, one from Northern, six from Upper East, four from Upper West, and four from Volta. All sequences were uploaded into GISAID and their accession numbers generated (Supplementary Table S1). Fourteen isolates were collected from LBMs, three from households, and one from a commercial farm. Phylogenetic analyses of Hemagglutinin (HA) genes confirmed that all LPAIV A(H9N2) detected in this study belonged to clade G1 (Figure 3). The viruses clustered closely with each other and with other LPAIV A(H9N2) from the West African region, especially those from neighboring countries such as Togo, Burkina Faso, and Nigeria (Figure 3).

### 3.5. Genetic Analyses of HA Gene on Successfully Sequenced LPAIV A(H9N2) Samples

All isolates possessed the amino acid motif RSSRGLF at the HA cleavage site. A Nucleotide Basic Local Alignment Search Tool (BLAST) (https://blast.ncbi.nlm.nih.gov/Blast.cgi (accessed on 16 June 2026)) analysis showed that the Ghanaian viruses shared high nucleotide sequence similarity (>90%) with a virus isolated from Togo in 2019 [A/chicken/Togo/EC-54/2019], with the highest similarity observed at 99.88%. Comparative analysis between the LPAIV A(H9N2) from Ghana and the Togo strain revealed several variations in the HA gene, including at the signal peptide (13T), receptor-binding domain (43T, 45K, 61N, 62Q, 72G, 78H, 95I, 104L), antigenic site (260G, 304G), and HA2 fusion domain (354I, 502E) (Table 3). None of the viruses harbored the H274Y mutation in the neuraminidase (NA) segment. Notably, all samples encoded key mammalian adaptation markers (15I, 54I, and 215A) in the matrix gene. Additionally, less-known mammalian host-specific markers in the PB2 gene (105V and 185I) were detected in four isolates.

## 4. Discussion

With the imminent threat posed by avian influenza viruses, surveillance continues to be the support system for early detection, control, and research. In Ghana, during the COVID-19 pandemic, outbreaks of AIVs among poultry were recorded. In view of this, the Noguchi Memorial Institute for Medical Research, in collaboration with the Veterinary Services Directorate and with support from the US Naval Medical Research Unit—EURAFCENT, conducted a country-wide surveillance among poultry, pigs, the environment, and farmers/LBM workers/animal handlers. We visited commercial farms, LBMs, backyard farms, and Ramsar sites for environmental samples.

In this study, we detected LPAIV A(H9N2) circulating predominantly among asymptomatic domestic birds, with a higher detection rate from LBMs compared to commercial farms and backyard farms. Logistic regression analyses using commercial farms as a reference showed a significantly reduced likelihood of detecting LPAIV A(H9N2) in backyard farms and increased odds for LBMs. This observation agrees with other studies conducted in Vietnam, where A(H9N2) was detected in LBMs all year round. This Vietnam study observed that LBMs were considered to be the epicenter for outbreaks, species-boundary crossing, and reassortment of avian influenza viruses [24]. A similar finding was reported in a Nigerian study in 2019, where LPAIV A(H9N2) was isolated from asymptomatic poultry in LBMs [25]. Our findings are consistent with previous reports identifying LBMs as the epicenter of avian influenza virus transmission, hence the need to intensify surveillance and preventive measures in this setting. Commercial and backyard farms also need to be closely monitored, as other Ghanaian studies have detected LPAIV A(H9N2) in these settings [17,18]. Inadequate biosafety and biosecurity measures, along with poor sanitation and insufficient ventilation, are factors that have been associated with conditions that facilitate antigenic shift and drift, rendering LBMs hotspots for avian influenza viruses [26]. The detection of avian influenza A(H9N2) in asymptomatic birds is alarming, as it poses a risk to individuals purchasing birds for consumption, who can be exposed to the virus during preparation. Similar risks apply to farmers/LBM workers/animal handlers and others near infected animals. Although no human cases were identified during this study, continued circulation of the virus in poultry warrants ongoing vigilance and risk mitigation measures.

LPAIV A(H9N2) was more prevalent in the Northern belt of Ghana compared to the Middle and Southern belts. Importantly, we observed a higher prevalence of the virus in guinea fowls compared to other types of poultry (Table 2). Guinea fowls are an abundant source of poultry for residents in Northern Ghana; so, infected guinea fowls could have food security implications. More cases among poultry (including guinea fowls) in Northern Ghana, could be attributed to porous borders, facilitating little to no restriction on bird importation in these regions [27]. In this context, the International Health Regulations (IHR, 2005) highlight the significance of introducing border surveillance, which enables port health units to detect pathogens of public health concern and implement timely mitigation measures [28]. Since 2016, avian influenza A(H9N2) has been circulating among poultry in Western and Northern Africa, demonstrating its persistence in the region [11]. Countries impacted by these outbreaks include Algeria, Burkina Faso, Morocco, and Togo. Notably, all these countries are located to the north of Ghana, except for Togo, located east of Ghana [11,29,30,31]. It is plausible that the viruses detected in this study were imported into the country through regional poultry movement. This endemic situation also seems to be a direct response to the global circulation of these viruses. The southern zone of Ghana, which includes the country’s coastline, contains four of the six designated Ramsar sites and serves as temporary settlement sites for migratory birds, which are primary reservoirs for avian viruses [32]. Although we did not sample migratory birds, interaction with domesticated birds has been known to mediate the transmission of the virus into the poultry population [25]. The detection of viruses in LBMs, together with their circulation in poultry across the West African region, might pose a significant threat to the sub-region’s food security and human health and underscores the need for sustained surveillance and coordinated infection control measures. Targeted interventions, such as improved monitoring of poultry movement, should be put in place as soon as possible to substantially reduce transmission risk.

Phylogenetic analyses of LPAIV A(H9N2) isolated from this study confirmed that the viruses belonged to the G1 clade, circulating among poultry in West and Northern Africa and clustered with each other and with viruses circulating in other West African countries, such as Togo, Burkina Faso, Senegal, Benin, and Nigeria. These G1 clade viruses are also known to be highly related to viruses circulating in Northern Africa and the Middle East [15,33,34,35].

Within the receptor-binding site in HA, genetic analyses showed amino acid substitutions at positions 191H, 197T, 198A, and 234L (H9 numbering), which are crucial for the transmission of avian A(H9N2) viruses via respiratory droplets in mammalian hosts, as shown in ferret models [36]. Notably, all Ghanaian isolates exhibited the 191H and 234L substitutions, which are associated with preferential binding to cellular receptors present in various human respiratory epithelial cells [37]. Six glycosylation sites located at positions 29, 105, 141, 298, 305, and 492 were found in most of the Ghanaian isolates. The glycosylation site at position 105 was present in all isolates except A/turkey/Upper West/AI-T-22-1569/2022, while the site at position 208 was found in all isolates except A/duck/Volta/AI-T-22-541/2022 and A/chicken/Ashanti/AI-T-22-856/2022. Furthermore, the site at position 305 was absent in A/duck/Volta/AI-T-22-541/2022 and A/guinea fowl/Upper West/AI-T-21-1529/2022. These glycosylation sites have been implicated in altering viral properties, such as immune evasion and host adaptation, which may impact pathogenicity and transmission dynamics of the virus in different bird species [36,38]. None of the viruses harbored the H274Y mutation in the neuraminidase (NA) segment, a well-characterized substitution known to confer resistance to the antiviral drug oseltamivir. All viruses encoded key mammalian adaptation markers in the matrix gene: 15I, 54I, and 215A, which have been associated with enhanced replication and increased human-to-human transmissibility [16,37]. Additionally, PB2 gene analysis identified the 105V substitution, linked to human-to-human virus transmission, and the mammalian-host-associated marker 185I, suggesting a potential risk for adaptation to the human host [39,40]. Low-pathogenic avian influenza viruses with the virulent-type sequence RXXR cleavage motif have the potential to evolve into highly pathogenic strains while circulating in chickens [41]. Mutations in the HA protein and the cleavage site can contribute to increased virulence and transmission efficiency, as shown by previous studies in both avian and mammalian models [36,38]. LPAIV of the A(H9N2) subtype has been known to cross the species barrier to infect humans. Although human infections with A(H9N2) remain rare, isolated cases have been reported in West Africa. The first case in West Africa was reported in Senegal, in a child [35], and, more recently, Ghana has also reported a crossover case in a child [42]. Continuous circulation among poultry in West Africa and in Ghana, especially in LBMs, represents a potential public health concern and highlights the importance of early detection and risk mitigation.

This study did not include ensnaring and direct sampling of wild birds for pathogen detection, which may contribute to the close relatedness between the Ghana and Togo H9N2 virus strains, beyond trade-related activities. They were, however, indirectly sampled by sampling bird feces, feathers and water samples at the RAMSAR sites.

In this study, we estimated the prevalence of LPAIV A(H9N2) to be 5%, with no significant association with the administrative zone, although the majority of positive birds were detected in the Northern belt of Ghana. The odds of detecting LPAIV in backyard farms and commercial farms are significantly lower compared to LBMs. Although no human case of LPAIV A(H9N2) had been recorded at the time of this study, it is expedient to continuously educate birdkeepers/farmers and conduct routine testing for both the birds and their keepers. The LPAIV A(H9N2) detected was closely related to H9N2 viruses circulating among poultry in other West African countries. The Ghanaian isolates possessed mammalian-adaptive features and the RXXR cleavage motif, increasing the potential to evolve into highly pathogenic strains. These findings highlight the importance of continuous surveillance as a pre-emptive strategy to strengthen monitoring of viral circulation, detect genetic changes, and support evidence-based preparedness efforts.

## Figures and Tables

**Table 1:** Demographic characteristics of birds, pigs, environmental, and farmer/LBM worker/animal handler specimens collected nationwide in 2022.

Variables	Animals Sampled	A(H9N2)-Positive	*p*-Value	AH3	SARS-CoV-2
	*N* = 1627	[*n* = 86, *n* (*n*/*N*)]		[*n* = 1]	[*n* = 1, *n* (*n*/*N*)]
**Animal type**					
Duck	163	5 (3.07)	# <0.0001	0 (0)	0 (0)
Fowl	1098	64 (5.83)		0 (0)	0 (0)
Guinea fowl	134	16 (11.94)		0 (0)	0 (0)
Turkey	69	1 (1.45)		0 (0)	0 (0)
Swine	111	0 (0)		0 (0)	0 (0)
Other birds	52	0 (0)		0 (0)	0 (0)
**Health Status**					
Dead	2	0 (0)	# 1.000	0 (0)	0 (0)
Healthy	1451	77 (5.31)		0 (0)	0 (0)
Sick	174	9 (5.17)		0 (0)	0 (0)
**Study settings**					
Backyard farm	907	3 (0.33)	<0.0001	0 (0)	0 (0)
Commercial farm	424	9 (2.12)		0 (0)	0 (0)
Live bird market	296	74 (25.00)		0 (0)	0 (0)
**Administrative zone**					
Middle belt	331	18 (5.44)	0.006	0 (0)	0 (0)
Northern Belt	521	40 (7.68)		0 (0)	0 (0)
Southern Belt	775	28 (3.61)		0 (0)	0 (0)
**Other Sources**					
Environment	44	0 (0)	-	0 (0)	0 (0)
Human	379	0 (0)		1 (0.26)	1 (0.26)

# Fisher’s exact test. Variable names are shown in bold, and non-bold text represents the corresponding values.

**Table 2:** Odds ratio of LPAIV A(H9N2) infection by study settings, bird type, and administrative zone.

Variables	Odds Ratio	*p*-Value	95% Confidence Interval	
**Study settings**				
Backyard farm	0.1530	0.005	0.4121	0.5682
Commercial Farm	(ref)	-	-	-
Live bird market	15.3704	<0.0001	7.5504	31.2896
**Bird type**				
Duck	0.3799	0.055	0.1412	1.0227
Fowl	(ref)	-	-	-
Guinea fowl	1.0656	0.854	0.5426	2.0925
Turkey	0.3895	0.376	0.0483	3.1404
Swine	1			
Other birds	1			
**Administrative zone**				
Middle belt	1.6886	0.131	0.8562	3.3303
Northern belt	(ref)	-	-	-
Southern belt	2.0280	0.026	1.0868	3.7842

Variable names are shown in bold, and non-bold text represents the corresponding values.

**Table 3:** Amino acid profiles at selected biologically important positions in the HA gene of Ghanaian A(H9N2) viruses isolated in 2022.

H9 Numbering	Receptor-Binding Site (RBS)									Cleavage Site	Glycosylation Sites					
Viruses	166	191	197	198	232	234	235	236	399	335–341	29–31	105	141	298	305	492
A/turkey/Volta/AI-C-22-523/2022	S	H	T	A	N	L	I	G	K	RSSRGLF	NST	NGT	NVT	NST	NIS	NGT
A/duck/Volta/AI-C-22-524/2022	S	H	T	A	N	L	N	G	K	RSSRGLF	NST	NGT	NVT	NST	NIS	NGT
A/chicken/Greater Accra/AI-T-22-0054/2022	S	H	T	A	N	L	I	G	K	RSSRGLF	NST	NGT	NVT	NST	NIS	NGT
A/chicken/Greater Accra/AI-T-22-0060/2022	S	H	T	A	N	L	I	G	K	RSSRGLF	NST	NGT	NVT	NST	NIS	NGT
A/duck/Volta/AI-T-22-524/2022	S	H	T	A	N	L	I	G	K	RSSRGLF	NST	NGT	NVT	NST	NIS	NGT
A/duck/Volta/AI-T-22-541/2022	S	H	T	A	N	L	I	G	K	RSSRGLF	NST	NGT	NVT	DST	NIN	NGT
A/chicken/Ashanti/AI-T-22-856/2022	S	H	T	A	N	L	I	G	K	RSSRGLF	NST	NGT	NVT	NSK	NIS	NGT
A/guinea fowl/Northern/AI-T-22-1150/2022	S	H	T	A	N	L	I	G	K	RSSRGLF	NST	NGT	NVT	NST	NIS	NGT
A/chicken/Upper East/AI-T-22-1384/2022	S	H	T	A	N	L	I	G	K	RSSRGLF	NST	NGT	NVT	NST	NIS	NGT
A/chicken/Upper East/AI-T-22-1406/2022	S	H	T	A	N	L	I	G	K	RSSRGLF	NST	NGT	NVT	NST	NIS	NGT
A/guinea fowl/Upper West/AI-T-21-1529/2022	S	H	T	A	N	L	I	G	K	RSSRGLF	NST	NGT	NVT	NST	NIN	NGT
A/turkey/Upper West/AI-T-22-1569/2022	S	H	T	A	N	L	N	G	K	RSSRGLF	NST	NGI	NVT	NST	NIS	NGT

## Data Availability

The data presented in this study are available from the corresponding author upon reasonable request. Public access to the data is restricted due to confidentiality, security and sensitivity considerations.

## Supplementary Materials

The following supporting information can be downloaded at: https://www.mdpi.com/article/10.3390/v18070725/s1, Table S1: Illustrates the assigned accession numbers to all A(H9N2) sequences uploaded into GISAID.
