# Supplementary material for: Genetic and Epidemiological Evidence of Avian Influenza A(H9N2) Detection Among Poultry in Ghana, 2022
Source: Viruses. 2026 Jun 30;18(7):725. doi: 10.3390/v18070725 (PMC13431622; doi:10.3390/v18070725)
Supplement: Supplementary file 1 [file viruses-18-00725-s001.zip › viruses-4319853-supplementary.pdf]

Supplementary

Table S1: illustrates the assigned accession numbers to all A(H9N2) sequences uploaded into GISAID

Accession number	Isolate Name	Originating Sample ID
EPI_ISL_18928498	A/Guinea Fowl/Northern/AI-T-22-1150/2022	AI-T-22-1150
EPI_ISL_18928494	A/Guinea Fowl/Upper East/AI-T-22-1344/2022	AI-T-22-1344
EPI_ISL_18928451	A/Guinea Fowl/Upper East/AI-T-22-1366/2022	AI-T-22-1366
EPI_ISL_18928432	A/Guinea Fowl/Greater Accra/AI-T-22-0858T/2022	AI-T-22-0858
EPI_ISL_18928406	A/Guinea Fowl/Upper West/AI-T-22-1527/2022	AI-T-22-1527
EPI_ISL_18928499	A/Chicken/North East/AI-T-22-1272T/2022	AI-T-22-1272
EPI_ISL_18928496	A/Chicken/North East/AI-T-22-1272/2022	AI-T-22-1272
EPI_ISL_18928495	A/Chicken/Northern/AI-T-22-1148/2022	AI-T-22-1148
EPI_ISL_18928453	A/Chicken/Northern/AI-T-22-1092T/2022	AI-T-22-1092
EPI_ISL_18928452	A/Chicken/Upper East/AI-T-22-1408/2022	AI-T-22-1408
EPI_ISL_18928434	A/Chicken/Upper East/AI-T-22-1440/2022	AI-T-22-1440
EPI_ISL_18928431	A/Chicken/Upper East/AI-T-22-1442/2022	AI-T-22-1442
EPI_ISL_18928429	A/Chicken/Greater Accra/AI-T-22-0857/2022	AI-T-22-0857
EPI_ISL_18928409	A/Chicken/Upper West/AI-T-22-1523/2022	AI-T-22-1523
EPI_ISL_18928364	A/Chicken/Ashanti/AI-T-22-0852/2022	AI-T-22-0852
EPI_ISL_18928362	A/Chicken/Ashanti/AI-T-22-0846/2022	AI-T-22-0846
EPI_ISL_18927568	A/Chicken/Upper West/AI-T-22-1530/2022	AI-T-22-1530
EPI_ISL_18927382	A/Chicken/Upper West/AI-T-22-1540/2022	AI-T-22-1540
EPI_ISL_18928243	A/Duck/Greater Accra/0058T/2022	AI-T-22-0058T
EPI_ISL_18636086	A/duck/Volta/AI-C-22-541/2022	AI-C-22-541
EPI_ISL_18636060	A/Duck/Volta/AI-C-22-524/2022	AI-C-22-524
EPI_ISL_18928497	A/Chicken/North East/AI-T-22-1273/2022	AI-T-22-1273
EPI_ISL_18928433	A/Chicken/Upper East/AI-T-1384/2022	AI-T-1384
EPI_ISL_18928430	A/Chicken/Upper East/AI-T-1395/2022	AI-T-1395
EPI_ISL_18928408	A/Chicken/Upper East/AI-T-22-1405/2022	AI-T-22-1405
EPI_ISL_18928399	A/Chicken/Upper East/AI-T-22-1406/2022	AI-T-22-1406
EPI_ISL_18928365	A/Chicken/Greater Accra/AI-T-22-0090/2022	AI-T-22-0090
EPI_ISL_18928363	A/Chicken/Greater Accra/AI-T-22-0086/2022	AI-T-22-0086
EPI_ISL_18928325	A/Guinea Fowl/Greater Accra/AI-T-22-0082/2022	AI-T-22-0082
EPI_ISL_18928249	A/Chicken/Greater Accra/AI-T-22-0063/2022	AI-T-22-0063
EPI_ISL_18928246	A/Chicken/Greater Accra/AI-T-0061/2022	AI-T-0061
EPI_ISL_18928245	A/Chicken/Greater Accra/AI-T-22-0060a/2022	AI-T-22-0060
EPI_ISL_18928244	A/Chicken/Greater Accra/AI-T-22-0060/2022	AI-T-22-0060
EPI_ISL_18927308	A/Chicken/Greater Accra/AI-T-22-0054/2022	AI-T-22-0054
EPI_ISL_18638133	A/chicken/Greater Accra/AI-T-22-0053/2022	AI-T-22-0053
EPI_ISL_18638132	A/chicken/Greater Accra/AI-T-22-0052/2022	AI-T-22-0052
EPI_ISL_18550128	A/chicken/Volta/AI-C-22-523/2022	AI-C-22-523
